# Lipoprotein glomerulopathy treated with LDL-apheresis (Heparin-induced Extracorporeal Lipoprotein Precipitation system): a case report

**DOI:** 10.1186/1752-1947-3-9311

**Published:** 2009-12-02

**Authors:** Gianpaolo Russi, Luciana Furci, Marco Leonelli, Riccardo Magistroni, Nicola Romano, Paolo Rivasi, Alberto Albertazzi

**Affiliations:** 1 Transfusion Medicine and Immunohaematology Unit, Azienda Ospedaliera S Maria Nuova di Reggio Emilia, Viale Risorgimento 80 42100 Reggio Emilia, Italy; 2 Nephrology, Dialysis and Transplantation Clinic, Azienda Ospedaliero-Universitaria Policlinico di Modena, Largo Del Pozzo 71 41100 Modena, Italy

## Abstract

**Introduction:**

Lipoprotein glomerulopathy is a glomerulonephritis which was described for the first time by Saito in 1989 and is currently acknowledged as a separate nosological entity. It is histologically characterized by a marked dilatation of the glomerular capillaries and the presence of lipoprotein thrombi in the glomerular lumens. The dyslipidemic profile is similar to that of type III dyslipoproteinemia with Apolipoprotein E values that are often high; proteinuria and renal dysfunction are present. Proteinuria often does not respond to steroid and cytostatic treatments.

The phenotypic expression of lipoprotein glomerulopathy is most probably correlated to a genetic alteration of the lipoprotein metabolism (mutation of the Apolipoprotein E coding gene). In literature, lipoprotein glomerulopathies have mainly been reported in Japanese and Chinese subjects, except for three cases in the Caucasian race, reported in France and the USA.

**Case presentation:**

We describe the case of a 60-year-old female, Caucasian patient suffering from lipoprotein glomerulopathy, carrier of a new mutation on the Apolipoprotein E gene (Apolipoprotein E_MODENA_), and treated successfully with low density lipoprotein-apheresis with the Heparin induced extracorporeal lipoprotein precipitation system. After a first phase of therapeutic protocol with statins, the patient was admitted for nephrotic syndrome, renal failure and hypertension. Since conventional treatment alone was not able to control dyslipidemia, aphaeretic treatment with heparin-induced Extracorporeal Lipoprotein Precipitation - apheresis (HELP-apheresis) was started to maintain angiotensin converting enzyme inhibitor therapy for the treatment of hypertension. Treatment with HELP-apheresis led to a complete remission of the proteinuria in a very short time (four months), as well as control of hypercholesterolemia and renal function recovery.

**Conclusion:**

According to this case of lipoprotein glomerulopathy, we believe that renal damage expressed by proteinuria correlates to the levels of lipids and, furthermore, the treatment with HELP-apheresis, by lowering low-density lipoprotein cholesterol and triglycerides, may be considered as a therapeutic option in synergy with pharmacological treatment in the treatment of lipoprotein glomerulopathy.

## Introduction

Glomerular damage is frequently caused by fat deposits in various anomalies of lipid metabolism (Fabry's disease, Fish Eye disease, von Gierke's disease, type III hyperlipoproteinemia), with a glomerulonephritis. This develops into glomerulosclerosis with severe nephrotic syndrome. From an initial asymptomatic proteinuria, chronic renal failure develops gradually as the atherosclerotic process advances.

The accumulation of lipids in the renal glomerule is a rare condition, described for the first time by Saito in 1989 [1] and currently acknowledged as a separate nosological entity with the term lipoprotein glomerulopathy (LPG).

LPG is histologically characterized by a marked dilatation of the glomerular capillaries and the presence of thrombi consisting of fat droplets in the dilated glomerular lumen. Staining with "Red-O oil" or Sudan red reveals the presence of these deposits with a "fingerprint" appearance on ultrastructural analysis. The presence of ApoE and ApoB can be shown in the thrombi using immunohistochemical methods. The lipid profile of patients suffering from this disease is similar to that of type III Hyperlipoproteinemia, with high levels of IDL and ApoE. Patients often have nephrotic proteinuria, but their lipid profile is different from that of subjects with nephrotic syndrome secondary to other kinds of glomerulonephritis. While patients with LPG have high levels of IDL, those with nephrotic syndrome due to other causes have high levels of LDL and VLDL.

The proteinuria in the nephrotic range often does not respond to steroids and cytostatic treatment.

Many studies attempted to find possible correlations between plasma levels, polymorphisms, mutations of ApoE and LPG with contradictory results. The phenotypic expression of LPG is probably correlated to a mutation of the coding gene for ApoE.

In the literature, LPGs have mainly been reported in Japanese and Chinese subjects, except for two cases in France and one in the USA, in Caucasian subjects [2,3].

## Case presentation

We describe the case of a 60-year-old Caucasian woman with a positive familial history for dyslipidemia and nephropathy.

The patient was first hospitalized in February 2001 with: hypertension (160/100 mmHg), high lipidic values (cholesterol 372 mg/dl, LDL cholesterol 267 mg/dl, triglycerides 239 mg/dl), creatininemia within the standard limits (0.9 mg/dl), a corresponding GFR of 63 ml/min calculated according to Cockroft and Gault, albuminemia of 3.15 gr/dl, total proteins of 5.7 g/dl, and a micro-hematuria, proteinuria (200 mg/dl) and proteinuria and/or albuminuria ratio of 2 g/g. The main immunological studies, ASO, rheumatoid factor, C3-C4, VDRL, serum immuno-fixation and serum immunoglobulin were normal. The serum markers for HBV, HCV and HIV were negative. The leucocyte formula was normal.

A physical examination ruled out the presence of xanthelasma and corneal arc in the absence of edemas. An ultrasound revealed kidneys which were regular in dimension and structure.

A renal biopsy showed the presence of lipid deposits in the glomerular lumens under the optical microscope after staining with Red-O oil. The interstitium showed a diffuse fibrous edema and tubular atrophy in the areas of fibrosis with a moderate infiltration of inflammatory cells. An arteriole showed hyaline deposits. An immunofluorescence revealed the presence of deposits of IgM and C_3 _in the sub endothelial seat. An electronic ultrastructural analysis confirmed the presence of thrombi in the lumen of the glomerular capillary loops. The histopathological diagnosis suspected LPG, but ApoE typing identified the polymorphism (E2/2) with a diagnosis of familial type III dyslipoproteinemia.

The patient started treatment with atorvastatin 40 mg/day. We did not use fibrates in association with statin to avoid possible side effects. After one year of therapy we observed a good control of lipid values (cholesterol 250 mg/dl, LDL cholesterol 114 mg/dl) and a reduction of the proteinuria (120 mg/dl). Hypertension was controlled (145/90 mmHg) by means of ACE-inhibitors (irbesartan 300 mg/day, ramipril 5 mg/day), beta-blockers (carvedilole 25 mg/day) and Ca antagonists (nifedipine 60 mg/day).

Four years later in April 2005, the patient was again hospitalized for clinically overt nephrotic syndrome with proteinuria (5 g/24 h) and renal failure (creatinine 2.2 mg/dl). Arterial hypertension (180/100 mmHg) was hardly responsive to treatment. The patient had a low response (cholesterolemia 445 mg/dl, LDL 318 mg/dl, triglycerides 292 mg/dl) to lipid-lowering drugs (atorvastatin 40 mg/day + cholestyramine 4 g/day + Omega3 fatty acids 2 g/day) with the presence of periocular thelasmas.

A renal biopsy revealed the presence of lipids with a fibrous growth of the interstitium and a percentage increase of sclerotic glomeruli with a diagnosis of LPG evolved into nephrotic syndrome (histology will be published in a manuscript in preparation and when published the reference will be provided).

In view of the patient's poor response to pharmacological treatment alone, in July 2005 we decided to associate for the first time in a patient with LPG an LDL-Apheresis protocol (three aphereses in the first two weeks, then one weekly session for two months followed by one apheresis every 15 days) with dextran sulfate columns on the basis of the evidence present in literature for the treatment of the steroid-resistant nephrotic syndrome. [4-6]

During the first LDL-apheresis (July 2005) with dextran sulphate columns (Liposorber System - Kaneka™), an anaphylactoid reaction occurred. This complication, as described by Olbricht in 1992 in patients undergoing treatment with ACE inhibitors and an LDL-apheresis regimen with dextran sulphate columns, is due to the inhibition of kininases that metabolize bradikynin [7].

To avoid stopping the anti-hypertension treatment with ACE-inhibitors we decided to use another LDL-apheresis procedure: the extracorporeal precipitation of LDL-cholesterol induced by heparin in acid pH (HELP System, B. Braun Avitum™ Melsungen Germany). This procedure does not involve complications linked to the use of ACE-inhibitors, following the same protocol previously described.

The effects of a single HELP-apheresis on vascular homeostasis is a simultaneous, drastic reduction within two hours of cholesterol (-52%), LDL-cholesterol (-56%), VLDL-cholesterol (-52%), oxLDL (-47%), Lp(a) (-55%), triglycerides (-50%), fibrinogen (-56%), thrombin (-55%), von Willebrand factor (-56%), factor V (-57%), factor VII (-35%), PCR (-56%), plasma viscosity (-14%), erythrocyte aggregability (-60%), and thrombocyte aggregability (-60%). Also reported in the long-term treatment was an increase of HDL-cholesterol (+14%), peripheral muscle oxygenation (+33-50%), coronary flow reserve (+14%), and cerebral CO_2 _reactivity (+14%) [8].

In the LDL/fibrinogen apheresis procedure (HELP-apheresis), the plasma is obtained by filtration of the whole blood through a 0.55 μm pore-size filter and then mixed continuously in a 1:1 ratio with a solution of a 0.2 M sodium acetate buffer (pH 4.85) containing 100 IU/ml (300,000 UI) of heparin. The pH of the plasma-buffer solution reaches 5.12 and, at this value, the heparin binds the LDL-cholesterol, Lp(a), fibrinogen and triglycerides forming aggregates. These aggregates precipitate and are retained by the precipitate filter. Precipitate filter is a 0.4 μm pore-size polycarbonate filter from which plasma free from LDL-cholesterol, Lp(a) and fibrinogen is obtained and then passed through an anion exchange filter (heparin absorber) to adsorb excess heparin. The last stage of the system (dialysis filter) restores the physiological pH of the plasma and the balance of the liquids, removing excess fluid by ultrafiltration. The liquid used for dialysis is a sterile solution with a bicarbonate concentration of 35 mmol/l. After the dialysis process, the plasma, purified of lipids and fibrinogen, is returned to the patient mixed with the haematic cell components.

We processed 3000 ml of plasma. That means about 1.4 patient plasma volumes in two hours, with HELP machine (Plasmat Futura^®^, B. Braun Avitum, Melsungen Germany) and a disposable kit. There were no side effects with the HELP. method, except that the patient had problematic vascular accesses leading to flow problems, So, it became necessary to modify some software parameters (most notably the "PA minimum" threshold; software version 2.06.01) to be able to use a 17G fistula needle for the blood withdrawal and a 20G cannula needle for the blood reinfusion during the procedure. With this configuration, the flows were relatively moderate for an LDL-apheresis (blood flow of 50 ml/min and plasma flow of 20 ml/min), to complete the treatment target of 3000 ml. After some procedures we proceeded with the use of an 18G fistula needle in re-entry, maintaining a 17G fistula needle for drawing, obtaining flows that were definitely higher (blood flow of 80-100 ml/min and plasma flows of 25-30 ml/min). By increasing the flows, there was a marked reduction in the duration of the individual procedure, super-imposable to that of a patient with good vascular accesses.

After two months of treatment with LDL-apheresis (10 procedures), the laboratory data showed a progressive increase of albuminemia (from 3.39 to 3.70 g/dl) with partial remission of proteinuria (from 3.3 to 2 expressed as urine protein to creatinine ratio. A ratio of 0.1 is normal - protein and creatinine are expressed in mg/dl); and a progressive decline in creatininemia (from 1.9 to 1.6 mg/dl). The patient's anemia was improving and her arterial pressure was well under control with a reduction of the pharmacological dose, compared to two months earlier. There was also a significant drop in the mean values of TG (from 285 to 231 mg/dl) and LDL-Cholesterol (from 178 to 158 mg/dl) with reduction of xanthelasma. Pre-apheresis fibrinogen and Lp(a) mean values did not show a significant reduction. Considering the patient's clinical condition improvement, we decided to reduce the statins dosage (from 40 to 20 mg/die) continuing LDL-apheresis protocol of one session every two weeks.

Four months after aphaeretic treatment (15 procedures), the patient's albuminemia values were maintained (3.70 g/dl), her creatinine (1.3 mg/dl) and proteinuria (ratio 1.3) were reduced and her arterial pressure was stabilized (120-80 mmHg). There was a further reduction of LDL-cholesterol (105 mg/dl) and TG (134 mg/dl) mean values, with a suspension of cholestyramine and Omega-3 fatty acids treatment and a further reduction of the xanthelasmas. The LDL-Apheresis session was reduced to one session every three weeks.

**Figure 1:**
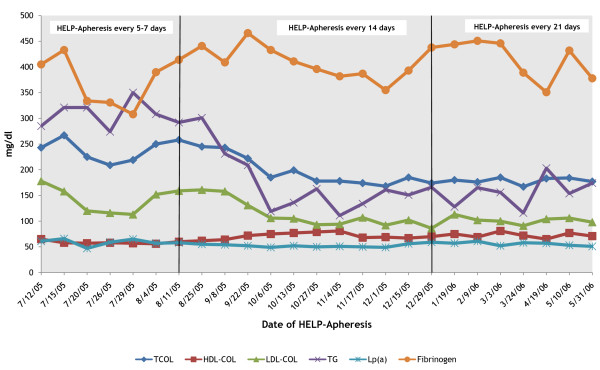
**Lipids and Fibrinogen values before every single H.E.L.P.-apheresis (first ten months of treatment)**. After the first ten months of apheretic treatment the mean values were: Total Cholesterol from 243 mg/dl to 184 mg/dl, HDL-Cholesterol from 65 mg/dl to 77 mg/dl, LDL-Cholesterol from 178 mg/dl to 106 mg/dl, Triglycerides from 285 mg/dl to 161 mg/dl, Fibrinogen from 405 mg/dl to 378 mg/dl, Lp(a) from 61 mg/dl to 51 mg/dl.

**Figure 2:**
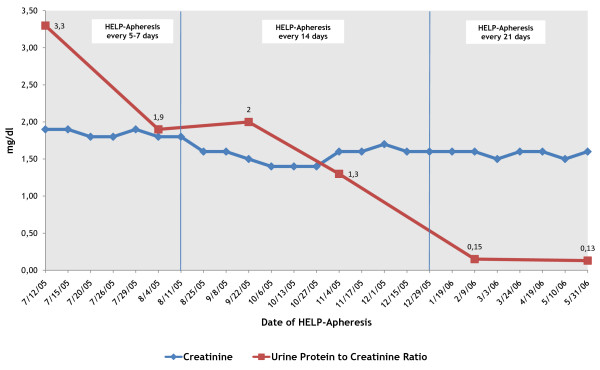
**Creatinine and Urine Protein to Creatinine Ratio before every H.E.L.P.-apheresis (first ten months of treatment)**. After the first ten months of apheretic treatment the mean values were: Creatinine from 1.9 mg/dl to 1.5 mg/dl and Urine Protein to Creatinine Ratio from 3.3 to 0.13 (protein and creatinine are expressed in mg/dl).

After ten months (25 procedures) the laboratory data were as follows: creatinine 1.6 mg/dl, albuminemia 4.30 g/dl, proteinuria (ratio 0.13), LDL-cholesterol 106 mg/dl and TG 154 mg/dl. The values of Lp(a) and fibrinogen are unchanged (Figure 1, 2).

After two years of follow up, with a maintenance treatment of one LDL-apheresis session every three weeks in the last period, the mean biochemical values were: cholesterol 199 mg/dl, LDL-cholesterol 110 mg/dl, HDL-cholesterol 76 mg/dl, TG 112 mg/dl, fibrinogen 381 mg/dl, creatininemia 1.5 mg/dl, Proteinuria ratio 0.9, arterial pressure 120-80 mmHg. The pharmacological therapy is unchanged.

The sequence of the ApoE gene made in 2007 showed that the patient was homozygous for epsilon-2 allele (polymorphism E2/2 with Cys112 and Cys158 in the mature protein) and heterozygous for a novel mutation in exon 4: c.502 C>T [Arg 150>Cys of mature protein]. The mutant Apo E (ApoE_MODENA_) expresses a new residue of Cys in place of an Arg. This new cysteine residue could form a disulphide bridge with the other Cys residue of the E2 isoform resulting in ApoE polymerization (dominant negative effect). This phenomenon is probably the cause of both dyslipidemia and lipid thrombi in the glomerular capillaries [9].

## Discussion

The limitations of this case report are due to its rarity (as far as we know it is the first case of LPG in Italy) as well as the difficulty to reach the diagnosis (the sequence of the ApoE gene in 2007 made it possible for a complete diagnosis of LPG to be confirmed). In July 2005, after the poor response of the patient to lipid-lowering drugs, we decided to start with an LDL-Apheresis protocol as possible treatment instead of ApoE and TG to stop the glomerular damage. In patients with familial hypercholesterolemia, kidneys are frequently damaged by fat deposits leading to glomerulonephritis which develops into glomerulosclerosis with severe nephrotic syndrome. The direct negative effects of lipids and pro-inflammatory markers on the renal arteries endothelium are due to the formation of free radicals because of oxidized LDL (oxLDL). This induces an inflammatory condition with an impairment of the endothelial function. There is glomerular damage and passive filtration of lipoproteins in the mesangium with active phagocytosis by a part of the mesangial cells and macrophages which, by releasing cytokines and growth factors, increase mesangial proliferation [10]. In patients refractory to conventional treatment (diet and statins), LDL-apheresis is a valid therapeutic tool to be associated with drugs to rapidly reduce the hematic lipid values [11] and improve renal function, thereby reducing the toxic effect [12].

LDL apheresis has been used in the treatment of steroid-resistant nephrotic syndromes [13,14]. It has been used to reduce the inflammatory condition linked to the production of cytokine (IL-8) by the macrophages activated and by the abnormal numbers of oxLDL [15]. In addition, it has also been used to improve renal hemodynamics by reducing the production of vasoconstrictor eicosanoids (Thromboxane A_2_) and increasing Prostaglandin I_2_. LDL apheresis has also been used to increase the response to steroid therapy by removing the VLDL, which induces a reduction of dose-dependent specific binding sites for the dexamethasone on smooth muscle cells [16].

A potential advantage of HELP-apheresis versus dextran sulfate columns in the treatment of LPG may be related to the use of high heparin dosages. Heparin may activate LPL and HTGL and easily remove lipoproteins rich of TG like VLDL and IDL that are generally elevated in LPG. Another potential advantage of HELP-apheresis versus dextran sulfate may be the dialysis circuit present in the HELP-Plasmat Futura machine. This plasmatic dialysis process, fundamental to restoring the plasmatic physiological pH and the balance of fluid, is also able to slightly correct impaired renal parameters such as creatinine, urea, and uric acid, as reported by Thiery [17]. Furthermore, a strong improvement of hemorheology (viscosity reduction), thanks to the simultaneous removal of lipids (LDL, VLDL, Lp(a), triglycerides), associated with the removal of coagulation factors (Fibrinogen, Factor V, Factor VIII, Willebrand Factor) and pro-inflammatory markers (PCR, endothelial adhesion molecules) may be obtained by HELP-apheresis [18,19].

## Conclusion

The experience in this case of LPG leads us to believe that the renal damage expressed by proteinuria correlates with the levels of lipids. The selective removal of LDL-cholesterol, Lp(a), TG and fibrinogen through HELP-apheresis and pharmacological treatment led to the clinical remission of proteinuria, thereby improving the renal function parameters (creatininemia and albuminemia). This clinical improvement may be also explained by considering the capability of the HELP dialysis circuit to correct impaired renal parameters. This specific tool probably makes HELP-apheresis a first choice apheretic treatment for drugs-resistant LPG or nephrotic syndrome. HELP-apheresis allowed the patient to maintain the use of the ACE-inhibitor and to reduce the administration of statins at a lower dosage.

Finally, HELP-apheresis may be considered a therapeutic option in synergy with pharmacological therapy in the treatment of LPG.

## Abbreviations

ApoE: Apolipoprotein E; ApoB: Apolipoprotein B; PG: Lipoprotein Glomerulopathy; HELP.: Heparin induced Extracorporeal Lipoprotein Precipitation; LPL: Lipoprotein Lipase; HTGL: Hepatic Triglyceride Lipase: TG: Triglycerides; ox-LDL: Oxidazed LDL; LDL: Low density lipoprotein.

## Consent

Written informed consent was obtained from the patient for publication of this case report and accompanying images. A copy of the written consent is available for review by the Editor-in-Chief of this journal.

## Competing interests

The authors declare that they have no competing interests.

## Authors' contributions

LF, ML, RM, AA performed the clinical work and made the diagnosis. RG, RN, PR performed the LDL-Apheretic therapy. RG, LF, ML, RM acquired, analyzed and interpreted the patient data and helped to draft the manuscript. RG reviewed the literature and drafted the manuscript. All authors read and approved the final manuscript.
